# Nanocosmeceuticals: facets and aspects

**DOI:** 10.2144/fsoa-2019-0109

**Published:** 2020-08-06

**Authors:** Vividha Dhapte-Pawar, Shivajirao Kadam, Shai Saptarsi, Prathmesh P Kenjale

**Affiliations:** 1 Department of Pharmaceutics, Bharati Vidyapeeth University, Poona College of Pharmacy, Erandwane, Pune, Maharashtra 411038, India; 2 Bharati Vidyapeeth University, Bharati Vidyapeeth Bhavan, Lal Bahadur Shastri Marg, Pune, India

**Keywords:** antimicrobials, cosmetics, nanocosmeceuticals, nanotechnology, regulations, skin, sunscreens, topical, toxicity, transdermal delivery

## Abstract

The global cosmetic market prized $532.43 billion USD in 2017 is expected to reach $805.61 billion USD by 2023, with a 7.14% compound annual growth rate. These figures have appealed to the cosmeceutical players for developing new and effective products containing advanced materials. Cosmetics incorporated with pharmaceutical actives, termed as ‘cosmeceuticals,’ are receiving an overwhelming response from cosmetic industry. Nowadays, the implementation of nanotechnology for enhanced effectiveness of cosmeceuticals is witnessing a huge success. These applications include remedies for hair damage, wrinkles, aging and skin dryness. Currently, there is a need to establish regulations and harmonized guidelines for nanotechnology-based products to assess their efficacy, safety and toxicity profiles. This review summarizes current development, applications, safety and regulations of nanocosmeceuticals.

## Lay abstract

Cosmetics are the most lucrative consumer products – always in demand by people of all age groups; from baby lotions to antiwrinkle creams. Cosmetics with therapeutically useful actives, formulated using nanotechnology are regarded as nanocosmeceuticals. These nanocosmeceuticals are becoming more popular in the cosmetic industry. Inspite of their high demand, the biggest challenge for nanocosmeceuticals lies in their short- and long-term safety issues, environmental concerns and regulatory hurdles.

## Graphical abstract

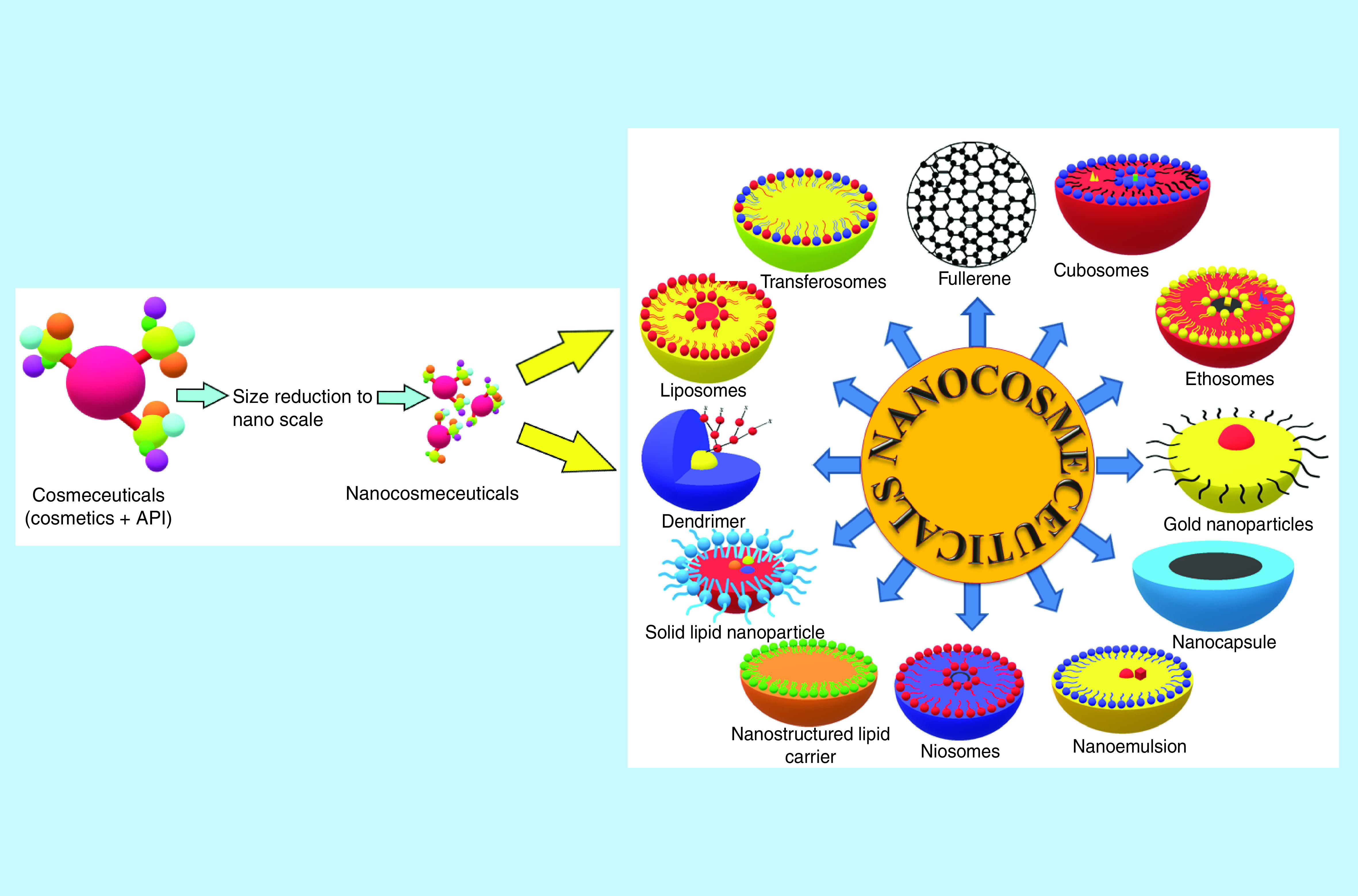

The US FDA has defined cosmetics as ‘substances intended for application to the human body aimed at cleansing, beautifying, promoting attractiveness or altering the appearance without affecting the body physiology or functions’ [1]. They are the popular consumer products in the global market; hence, it is a tempting area for various market players [2,3]. New modified lifestyles, unpredictable climatic changes and pollution have resulted in an increased need and supply of the cosmetics. The worldwide cosmetic market, valued at $532.43 billion USD in 2017 is likely to increase to $805.61 billion USD by 2023, with a 7.14% compound annual growth rate. According to a recent survey, Middle East and African regions are anticipated to rise in the cosmetic product market with the highest compound annual growth rate of 21% [2].

The worldwide rise in the aged population is due to reducing mortality rates. High demand of anti-aging cosmetic products globally has increased progressively due to profound need of men and women to look young. The estimated total number of people above 60 years of age will be 2.09 billion by the year 2050. Therefore, the demand for cosmetic products by elderly people will rise. Increased awareness for natural and herbal beauty products have created promising chances for companies to invent and design new products for cosmetic use [4,5]. Competitive market, advancing technology and the escalating demand for cosmetics have drawn company’s interest in continuous R&D in the personal care industry. Cosmeceuticals is one such impending application wherein the contemporary personal care products possess an active pharmaceutical ingredient with added therapeutic effectiveness. The term ‘cosmeceutical’ was coined by Raymond Reed and investigated by Dr Albert Kligman in the late 1970s [6].

Nowadays, cosmeceuticals are employed routinely in various circumstances, so as to improve or prevent wrinkles, skin dryness, dark spots, uneven complexion, hyperpigmentation, photo ageing and hair damage.

In recent years, cosmetics have emerged as the fastest flourishing field in the personal care products industry [7]. This field has amplified the treatment arena for medical practitioners to treat patients associated with skin disorders. At present, nanotechnology is explored in this cosmetic industry with a wide array of possible applications [8–10]. Cosmetic preparations comprising of the nanoparticles were termed as nanocosmetics (Figure 1) by Christian Dior in 1986. These products received attention when L’Oréal in 2005 discovered the commercial benefits of nanocosmetics [11]. After 2012, the FDA objected advertisements of the skincare products by L’Oreal as they would describe them as active pharmaceutical ingredients. This was followed by another FDA warning in 2015 against improper marketing and false therapeutic claims made by similar company in regard to its cosmetics products. In 2017, the FDA evaluated the corrective actions adopted by L’Oreal with an assurance of sustained compliance [12]. Currently, various countries have their own guidelines to regulate the cosmetic and cosmeceutical products. The guidelines must be reformed by considering the application, demand and effectiveness of nanotechnology driven cosmeceuticals. This review summarizes the current regulatory scenario which would be useful to make the harmonized guidelines that can be accepted worldwide. Also, this review will be helpful to the people serving in commercial and regulatory set-ups.

**Figure 1.**
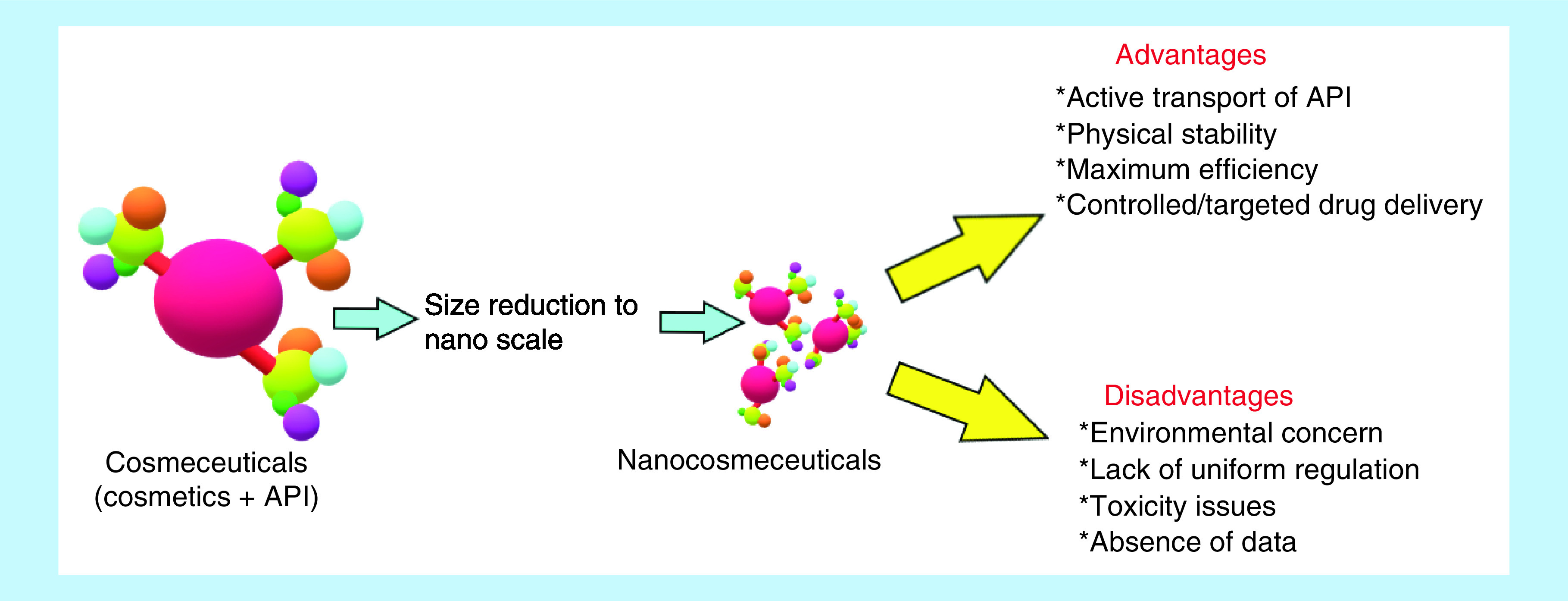
Nanocosmeceuticals – concept, advantages and disadvantages. API: Active Pharmaceutical Ingredient.

## Role of human skin in drug delivery

**Figure 2.**
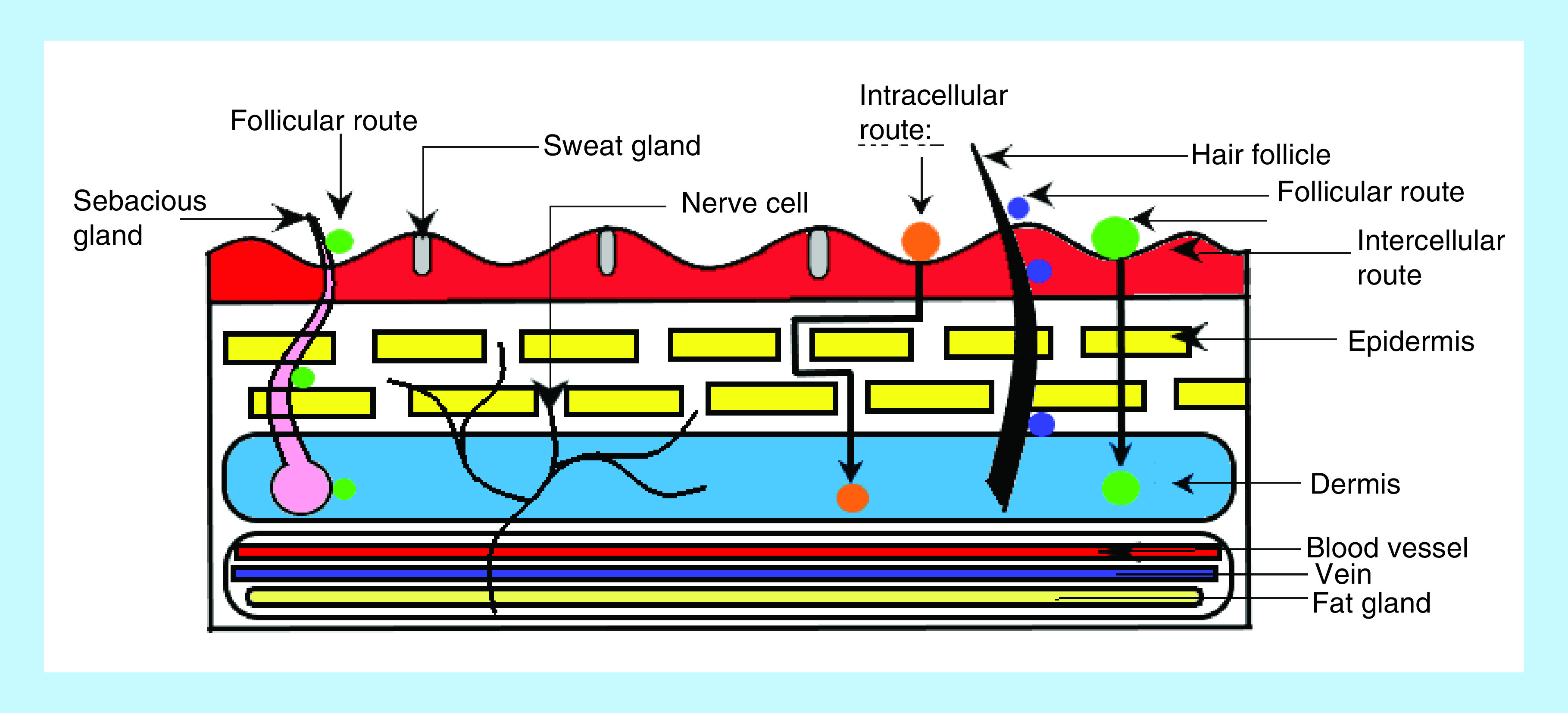
Possible transport pathways in human skin.

Human skin comprises major part of the body due to its high surface area. Skin is responsible for the protection, regulation as well as sensation. The average surface area of human skin is approximately 1.6–1.9 m^2^ [7,13]. Dermis, epidermis and hypodermis are the three main components of human skin [14–16] as seen in Figure 2.

## Possible mechanisms of action for nanocosmeceuticals

The stratum corneum (SC) layer of human skin as a permeation barrier offers an exclusive delivery pathway for the therapeutic agents as well as the cosmeceuticals. In topical and transdermal delivery, the active moieties may penetrate via the intercellular, intracellular and transappendageal routes either into the skin matrix or the subcutaneous tissues and subsequently into the systemic circulation, respectively [17,18]. The nano-sized carriers either can translocate without degradation inside the skin or can be degraded near the skin surface wherein the encapsulated therapeutic moieties would penetrate into the skin layers. At times, when most of the sub-micron sized particles are unable to cross the SC layer, the transappendageal route appears to be the dominant pathway for the entry of nanoparticles into the skin (Figure 2). Additionally, cutaneous penetration of the inorganic and polymeric nanoparticles can be facilitated using various passive and active permeation enhancement methods. The size, morphology, surface features, physicochemical properties of the nanomaterial along with their drug loading efficiency and lamellar arrangement defines the interactions between nanoparticles and skin to outline their delivery routes [18]. Lipid-based vesicular drug-delivery systems are preferred for the skin ailments. The polymeric nanoparticles comprising sunscreen agents permeate into the SC layer to deliver the encapsulated drug into the skin [19]. Inorganic nanoparticles like zinc oxide (ZnO) and titanium dioxide (TiO_2_) are prime examples of the transparent and cosmetically desirable sunscreens [20]. Metallic/magnetic nanoparticles are useful in cell labeling or skin targeting for early diagnosis of skin ailments. They can passively penetrate the SC layer and hair follicles, reaching up to the stratum granulosum [21]. Quantum dot nanoparticles preferentially accumulate in the hair follicles as well as the upper SC layers. They can penetrate the skin through SC intracellular lipid layers [22].

## Nanocosmeceuticals

**Figure 3.**
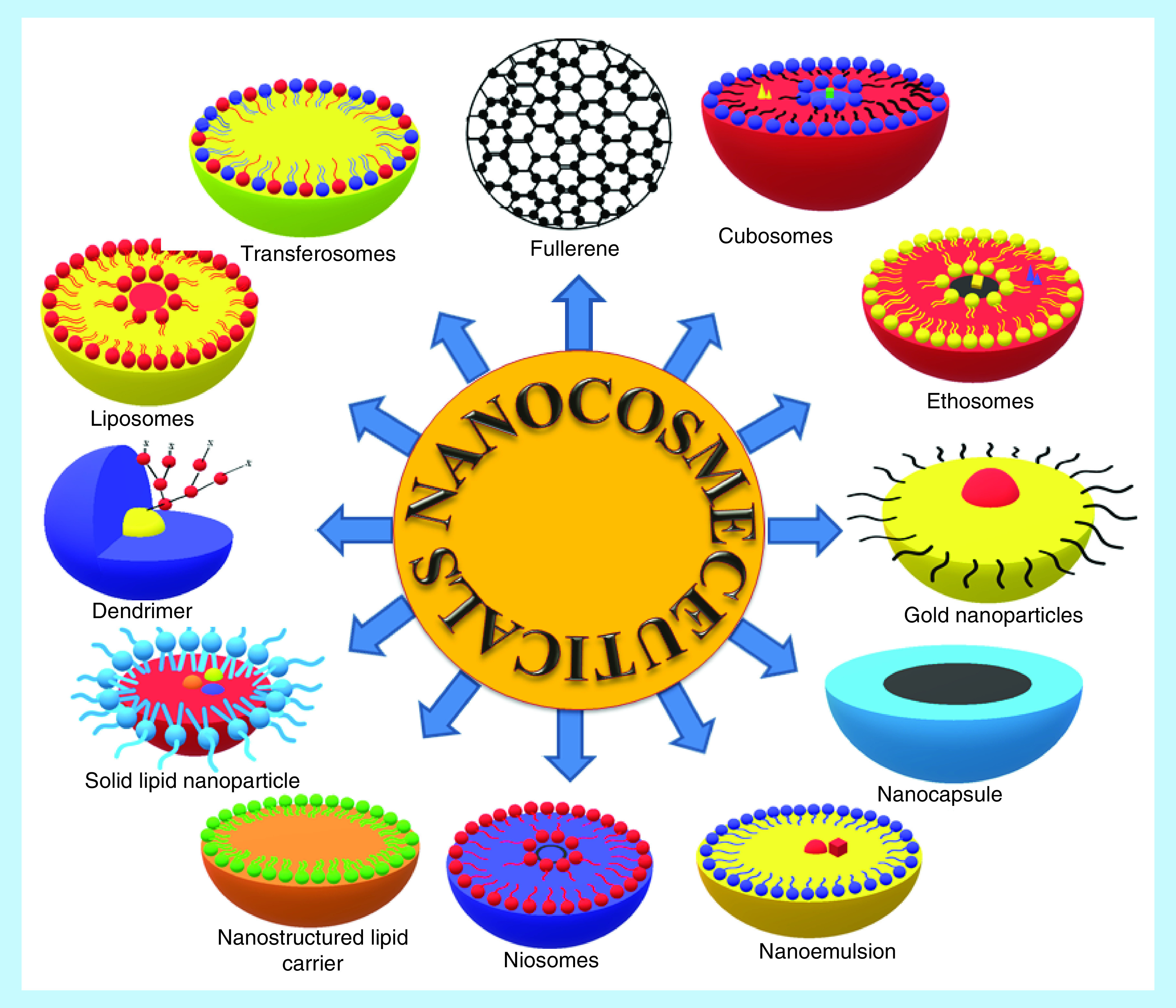
Types of nanocosmeceuticals.

Nanoparticles are the sub-micron sized particles in the size range of 1–100 nm (Figure 3) [23,24]. Nanoscience and nanotechnology explores the distinct traits of the materials observed in nanosize. These remarkable traits have reformed and transformed each and every field of basic and applied sciences [25]. Over the last three decades, nanoparticle-based technology is providing novel solutions to the treatment challenges associated with numerous acute and chronic ailments [26]. The nanoparticulate systems offer targeted delivery, painless remedies, customized therapies and simplified solutions over the conventional therapies [27]. Diverse forms of novel, sub-micron sized drug-delivery systems used in the delivery of cosmeceutical preparations comprise liposomes, nanostructured lipid carriers, solid lipid nanoparticles, niosomes, transferosomes, ethosomes, nanocapsules, nanoemulsions, gold nanoparticles, fullerenes, dendrimers and cubosomes (Figure 3). These novel forms have been successfully developed and commercialized [27,28]. Nowadays, the use of nanotechnology is increasing steadily in the personal care industry with better entrapment, dispersibility, performance, quality, protection, penetration and patient compliance [29,30].

Liposomes were one of the first colloidal vesicular systems (consisting of single or multiple lipid phosphatidylcholine bilayers) explored in effective skin delivery. Modified lipid-based nano-sized delivery systems such as nanostructured lipid carriers, solid lipid nanoparticles, transferosomes, niosomes and ethosomes have been developed to enhance the skin permeation of various active molecules [18]. Jean *et al.* investigated stable, transparent, oil-in-water nanoemulsion for hair care [30,31]. This nanoemulsion was prepared using nonionic emulsifying agents and consistency increasing agents like ceteareth-20, ceteareth-12, glyceryl stearate, cetyl alcohol and cetyl palmitate. This nanoemulsion containing heat-sensitive active agents demonstrated a mean particle size in the range of 50–200 nm [31]. Alain *et al.* prepared stable nanoemulsion using an amphiphilic lipid system, comprising ester or a mixture of esters [32]. Gunjan *et al.* prepared a cosmetic antiwrinkle cream containing extract of *Curcuma longa-*loaded ethosomes. The developed, 216 nm sized ethosomes helped in good dermal penetration of the extract along with the hydrating and moisturizing effects [33]. Similar studies were carried using transfersomes [34]. Cauchard *et al.* patented a cosmetic formulation, which released the active agent, an amber extract (0.01 and 5% w/w) in a programmed manner for improving the skin firmness [35,36]. Similarly, many nanocosmetics have been commercialized and readily available in the market (Table 1).

**Table 1.** Commercially available nanocosmeceutical formulations with their uses.

	Product	Company	Use
**A. Liposome**			
1.	Capture Totale^®^	Dior	Antiwrinkle effect help in removing blemish and dark spots
2.	Advanced Night Repair Protective Recovery Complex	Estee Lauder	As skin repairing agent
3.	Clinicians Complex Liposome Face and Neck Lotion	Clinicians Complex	Prevent ageing and help to provide nutrient to the skin
4.	Kerstin Florian Rehydrating Liposome day Creme	Kerstin Florian	Rehydrate the skin and acts as moisturizer
5.	Dermosome^®^	Microfluidics	Retaining and prevent loss of moisture from the skin
6.	Decorte Moisture Liposome Eye Cream	Decorte	Whitening the black skin around the eyes and also help in moisturizing.
**B. Niosome**			
1.	Niosome+	Lancome	Whitening skin and increase facial look
2.	Niosome+ Perfected Age Treatment	Lancome	Removing wrinkle and also help in skin cleansing
3.	MayuNiosome Base Cream	Laon Cosmetics	Moisturizing and whitening
4.	Anti-Age Response Cream	Simply Man Match	Antiwrinkle agent
5.	EusuNiosomeMakamPom Whitening Facial Cream	Eusu	Removing and decreasing wrinkle formation
6.	Niosome+	Lancome	Whitening skin and increase facial look
**C. Solid lipid nanoparticles**			
1.	Allure Body Cream	Chanel	Moisturizing the skin and body
2.	Soosion Facial Lifting Cream SLN technology	Soosion	Antiwrinkle agent and help in nourishing the skin
3.	Phyto NLC Active Cell Repair	SirechEmas	Rejuvenating skin and help in nourishing with decrease in pigmentation
**D. Nanoemulsion**			
1.	Vital Nanoemulsions A-VC	Marie Louise	Nourishing skin and miniaturization
2.	Precision-Solution Destressante Solution Nano Emulsion Peaux Sensitivity	Chanel	Moisturizing the skin.
3.	Coni Hyaluronic Acid and Nanoemulsion Intensive Hydration Toner	Coni Beauty	Hydrating skin and moisture retention
4.	Phyto-Endorphin Hand Cream	Rhonda Allison	Sooth and moisturize the skin
5.	Vitacos Vita-Herb Nona-Vital Skin Toner	Vitacos Cosmetics	Moisturizing the skin
**E. Gold nanoparticles**			
1.	ChantecailleNano Gold Enerizing Eye Serum	Chantecaille	Reducing the ageing process by increasing cell growth
2.	AmeiziiNano Gold Foil Liquid	Ameizii	Repairing damaged skin and moisturizing it with increased fairness
3.	LR Nano Gold and Silk Day Cream	LR Zeitgard	Protect and prevent skin cancer by preventing harmful radiation of sun
4.	O3+ 24 K Gold Gel Cream	O3+	Providing a glowing skin and provide shine
5.	Orogold 24K Nano Ultra Silk Serum	Orogold	Prevent moisture loss and keep a healthy skin
**F. Nanosphere**			
1.	Fresh As A Daisy Body Lotion	Kara Vita	Moisturizing the skin and also prevent water loss
2.	Hydralane Ultra Moisturizing Day Cream	Hydralane Paris	Moisturizing agent and in retaining moisture inside skin
3.	Clear It! Complex Mist	Kara Vita	Help to reduce pimples and prevent acne formation
4.	Cell Act DNA Filler Intense Cream	Cell Act Switzerland	Antiwrinkle agent
5.	Nanosphere Plus	Dermaswiss	Potential anti-aging agent

## Current regulations for nanocosmeceuticals

In these past years, there are many challenges witnessed in the regulation of the nanoparticulate systems. Numerous studies have been conducted to assess the discrepancies related to the safety of nanomaterials in cosmetics.

### FDA

The FDA regulates a large number of products by Federal Food, Drug and Cosmetic Act. Cosmetic products are not directly regulated by the FDA [36]. Currently, cosmetics do not need any prior approval, but efforts must be undertaken to assure safety of cosmetics. It is the manufacturer who must be accountable for ensuring the safety of cosmetic products. In 2006, the FDA created internal nanotechnology task force to regulate the nanoparticle-based products. This step was taken to boost the development of innovative, safe and effective nanomaterials in cosmetic and pharmaceutical sectors [37]. In 2007, amendments were suggested by the FDA; numerous have been executed and several are in pipeline. In 2014, FDA issued three guidelines related to the safety of the nanoparticulate systems in the FDA-regulated products; two of these are associated with the cosmetics. The first guideline explained the determination of FDA-regulated products containing nanoparticles, based on the particle dimensions and the dimension-dependent phenomena. The second guideline addressed the safety of nanomaterials in cosmetic products. It is not made mandatory by the FDA to reveal the nanomaterials used for formulation on the finished product label [38]. The FDA in association with the Personal Care Products Council (DC, USA) devised numerous regulations for cosmetics, based on the Voluntary Cosmetic Registration Program for voluntary reporting of the ingredients and the adverse reactions [39,40]. The FDA constantly updates the manufacturers about the risks related to nanomaterials for continuous improvement in the safety of the cosmetic products. With this initiative, the manufactures are also benefited as they can restrict the use of unsafe nanocosmeceuticals [41].

### EU

The EU regulations define nanomaterials as an insoluble or bio-persistent and intentionally manufactured material with one or more external dimensions, or an internal structure on the scale of 1–100 nm. It is necessary that all the nanomaterials must be indicated with the specific word ‘nano’ [42]. The information on product specification, toxicity, safety profile and undesirable effects needs to be informed 6 months prior to the market entry of nanoparticle-based products/nanocosmeceuticals. Premarket approval is required for the nanotechnology-derived sunscreens, colorants, cosmeceuticals and anti-aging products. Nanomaterials are covered under the European Commission Regulation Number 1907/2006, Registration, Evaluation, Authorization and Restriction of Chemical substances and Scientific Committee on Consumer Products safety [42–44].

### India

The Government of India judiciously raised funds in the Nano Science and Technology Initiative, which provided well-organized set-ups in various universities, academic societies, national laboratories, start-ups as well as R&D units. The key performers in the national health research systems are the Council of Scientific and Industrial Research, Indian Council of Medical Research, Department of Science and Technology and Department of Biotechnology (all in New Delhi, India). The Ministry of Health and Family Welfare (New Delhi, India), plays a pivotal role in the prevention and control of the health-related hazards. Additionally, the Nanotechnology Sectional Committee, which involves experts affiliated to varied research institutes and organizations, work actively for the standardization of the nanotechnology-based products [41].

### Other cosmeceutical regulations

Additional cosmeceutical regulations across various countries are summarized in the Table 2 [10,36,41–43].

**Table 2.** Cosmeceutical regulations in various countries.

	Country	Cosmeceuticals category	Highlights
1.	USA	Drug, cosmetic or Both	US FDA scrutinizes stringently those cosmeceuticals which alter the physiological processes in the skin. Cosmeceuticals with specified claims, aesthetic and functional benefits without clinical trials are not required to undergo the expensive and complicated FDA approval process
2.	Japan	Quasi-drugs	The ingredients which is to be used in quasi-drugs should be preapproved before selling and marketing
3.	Korea	Functional cosmetics	The Korea Food and Drug Administration plays an important role in improving safety and evaluation of functional cosmetics
4.	Thailand	Controlled cosmetics	Controlled cosmetics necessitate controlled ingredients for being marketed with the notification from FDA
5.	Canada	Dermo-cosmetics	Contains category V to accommodate product requirements for regulation
6.	Australia	Therapeutic goods	Only approved ingredients should be used for the manufacturing of these products
7.	China	Cosmetics for special use	Cosmetics have to be evaluated for safety and health quality test such as microbiology, toxicology test, chronic toxicity, carcinogenic test and conducting safe-for-human-use trials.

### Nanocosmeceutical applications

**Figure 4.**
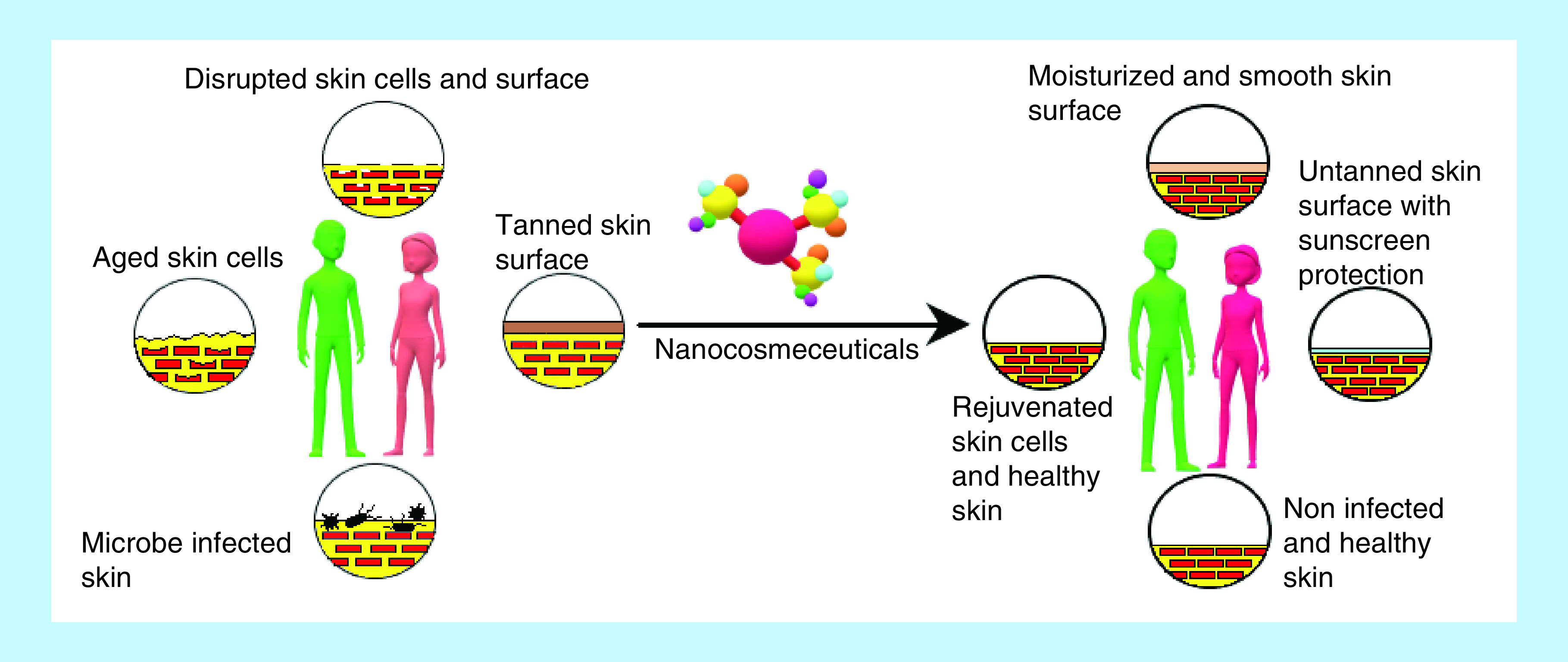
Major applications of nanocosmeceuticals.

Major applications of the nanocosmeceuticals are summarized in Figure 4 and discussed in the following sections.

#### Sunscreens

The prime use of sunscreens is to protect the skin from the short-term and long-term effects of UV radiation. The best sunscreen has a sun protection factor (commonly known as SPF) of at least 30 [42]. Nanoparticles used in the sunscreen cosmeceuticals do not form the typical, white, chalky layer on the skin as that of the conventional sunscreens. Nevertheless, the amount of established, sunscreen products applied on the skin can be minimized to a large extent with the help of nanoparticles. They also offer beneficial effects such as a less greasy appearance and odorless nature, which increase their aesthetic appeal [45]. Most used nanoparticles in sunscreen are TiO_2_, ZnO and para-aminobenzoic acid nanoparticles, which protect the skin from UV-A and UV-B rays [42,46].

#### Anti-aging products

Nanoparticle technology is most extensively utilized in anti-aging products. A very common anti-aging product which has a significant presence in the market is Revitalift^®^, manufactured by L’Oreal Company (Clichy, France). Revitalift uses nanosomes composed of pro-retinol A. The majority of anti-aging products work by moisturizing, lifting, toning and whitening the skin. Retinol A is known to increase the epidermal water content, epidermal hyperplasia with an enhanced collagen synthesis [47]. Additionally, retinol affects melanogenesis and inhibits the matrix metalloproteinase, which are responsible for collagen breakdown. Another example is Lancôme (Paris, France), who introduced Hydrazen Cream^®^, which is a nano-encapsulated triceramide formulation that rejuvenates skin health [48].

#### Moisturizer

Moisturizers are the most widely used cosmetic product, which also makes use of nanoparticles [49,50]. Water from the SC gets easily evaporated causing dryness and cracks. This can be treated with the help of the nanoparticles that form a thin humectant layer to prevent the rapid evaporation of water. Thus, the moisture content is retained resulting into better skin appearance [51]. Liposomes, nanoemulsions and solid lipid nanoparticles are the most widely used nanoparticulate in moisturizing formulations. They are useful in the treatment skin related diseases like dermatitis, psoriasis and pruritus [52].

#### Hair care products

The utilization of nanoparticles in the global hair care industry has recently emerged as an alternative to provide a better appearance and nutrition to the hair follicles. Additively, the use of nanoparticles in hair industry mainly focuses on the prophylaxis and the treatment of various types of alopecia [53]. Earlier, the common use of shampoos was restricted to cleansing only. Nowadays, nanoparticle-based shampoos are used as replacement therapies to provide nutrition essential for the proper growth of hair follicles. Silicone oil-based nanoparticles are used to diffuse inside the hair fibers without destroying their cuticle. This formulation offers more moisture and silkiness, as well as strengthens the hair owing to its nano size [54]. Nano size enables the penetration of silicone oil into the hydrolipid emulsion layer and prevents its accumulation on scalp.

## Toxicity, safety & hazardous effects of nanocosmeceuticals

### Toxicity associated with nanocosmeceuticals

There is a continuous, rapid growth in the novel technologies and pharmaceutical preparations related to the nanoparticles. Along with these advances, there is also a risk factor associated with the development procedure and use of the nano-based products in society. Toxicity issues are the major limitations of nanotechnology, alongside technological and economic barriers [54]. In the past few years, a large number of case studies such as cell uptake, genotoxicity and oxidative cell damage have raised issues related to the safety of nanocosmeceuticals [55]. The particle size on the scale of nano range makes the surface of particles highly active. Penetration of such highly surface-active nanoparticles via the skin can lead to adverse reactions [56].

Nano-sized TiO_2_ or ZnO (30–150 nm), up to 25% in concentration for the use in topical cosmetic products or sunscreens, are established as safe for protecting the skin from harmful solar radiations. Several reports have demonstrated that neither TiO_2_ nor ZnO nanoparticles can penetrate beyond the SC layer of skin under different test conditions [57]. When explorative studies were performed on the gold nanoparticles, they were discovered to be safe with negligible toxicity. Later on, the changes in size of gold nanoparticles demonstrated potential toxicity. Nanoparticles, below 10 nm size, can easily penetrate deep inside the skin layer and nuclear membrane leading to potential genotoxicity. Further, nanoparticles in cosmetic products should be prepared considering the toxicity initiating parameters like morphology and concentration.

Despite the possible challenges associated with nanoparticles, it has a wide array of significant applications [58]. Fullerene nanoparticles are researched for diverse dermatological applications such as antiacne, anti-aging, brightening essence and sunscreens, owing to their excellent antioxidant properties [59]. Numerous case studies have highlighted that the carbon fullerene-based cosmeceuticals can cause brain damage in fishes [60,61]. In a recent study by Dhawan *et al.* the colloidal, C60 fullerenes have demonstrated genotoxicity [62].

### Safety of the nanocosmeceuticals

**Figure 5.**
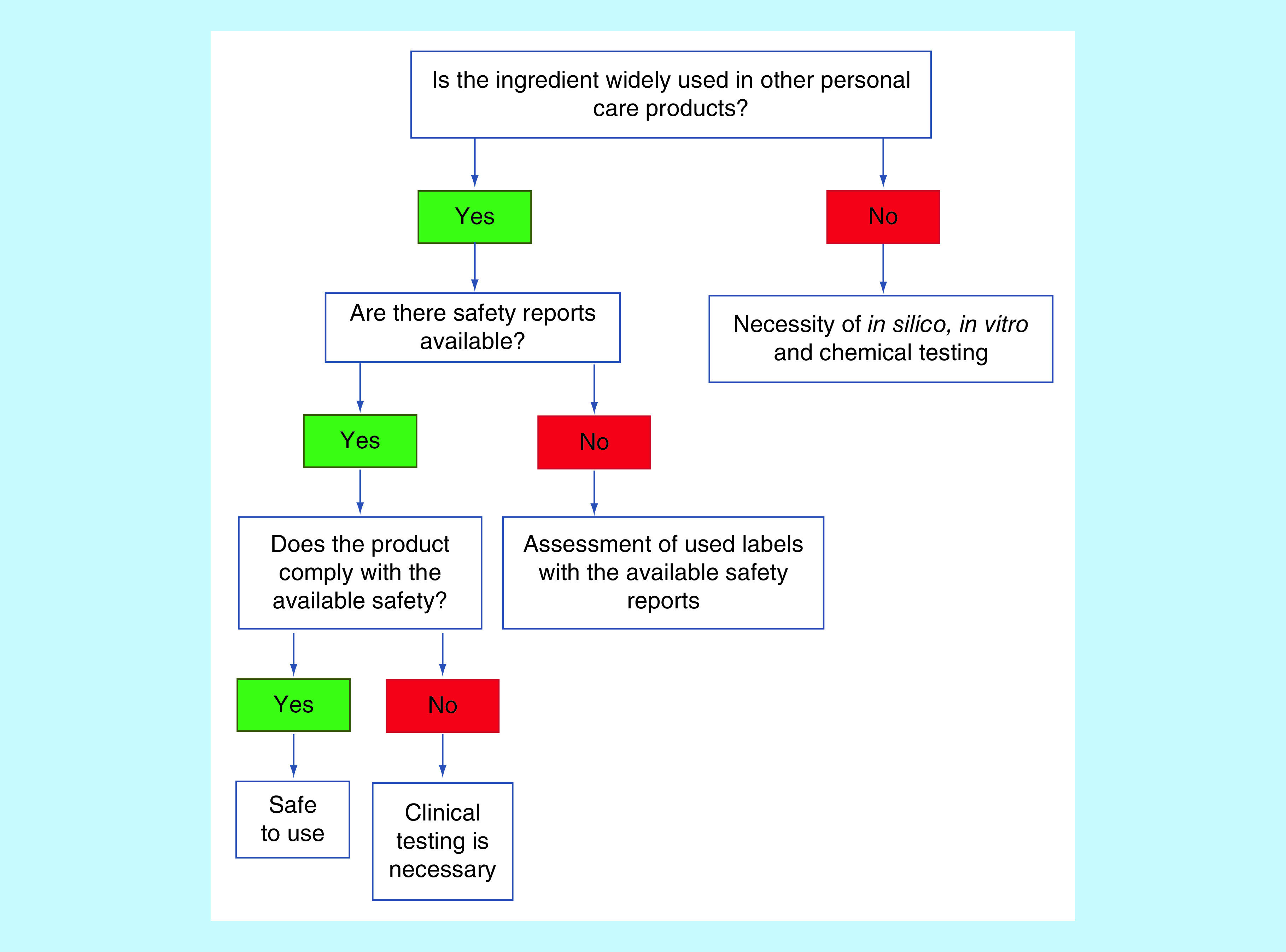
Decision making tree for safety evaluation of nanocosmeceuticals products.

As per the Scientific Committee on Consumer Products safety and European Commission, the safety of the cosmeceutical products is governed by the safety of their ingredients [63]. On the other hand, the FDA does not require cosmetic products and their ingredients to have FDA approval before marketing. The FDA states that the manufacturers can explore the safety data of the specific ingredients or products with comparable formulations to judge the safety of nanocosmeceuticals [64]. Likewise, the Personal Care Products Council measures and compares the safety of nanocosmeceuticals with the previously marketed formulations [65]. Individual ingredients safety facts and data are accessible through various resources such as the FDA’s Generally Recognized As Safe database and the European Commission’s Cosmetic Ingredient Database. An ingredient is grouped under Generally Recognized As Safe by a panel of qualified experts after being classed as ‘having adequate safety under the conditions of its intended use’ [66]. Even though this database mainly evaluates the ingredients such as the food additives, it also provides valuable safety inputs on the ingredients present in the personal care, as well as cosmetic products. Besides, the Cosmetic Ingredient Review (DC, USA) is an independent body involved in evaluating the safety of the individual ingredients used in cosmetics. Cosmetic Ingredient Review evaluates the ingredients using standardized procedures and specifies the recommendations as shown in Figure 5 [67].

#### Methods for assessing safety of the nanocosmeceuticals products

The active Scientific Committee on Emerging and Newly Identified Health Risks (Brussels, Belgium) controls and regulates the risk assessment methodologies, accessible for measuring the adverse health effects of the nanotechnology-based products [68]. The key factors that are judged for the safety of the nanomaterials are summarized in Table 3 [56,58].

**Table 3.** Evaluation parameters for the safety of nanomaterials.

	Parameter	Details
1	Physical–chemical properties	Particle size, particle morphology, surface area, pore diameter, agglomeration behavior, solubility and other chemical properties like molecular formula, chemical structure, final composition of nanomaterial, phase distinctiveness and hydrophilic–lipophilic nature
2	Mathematical modeling	Simple, empirical algorithms to complex mathematical equations
3	Microscopic techniques	Particle induced x-ray emission, laser scanning confocal microscopy, radio labeling with positron emitter ^48^V, high-resolution transmission electron microscopy
4	*In vitro* methods	Dermal absorption measurements on human/pig skin, phototoxicity testing via the 3T3 NPRT, MM and WEC, *in vitro* mammalian cell gene mutation test, genotoxicity/mutagenicity testing, Episkin™ or Epiderm™, *in vitro* micronucleus test or *in vitro* mammalian chromosome aberration test, skin corrosion testing via TER, embryotoxicity testing via three tests EST, skin irritation testing via Episkin

EST: Embryonal stem cell test; MM: Micromass assay; TER: Transcutaneous electrical resistance; WEC: Whole embryo culture.

## Conclusion

There is always a growing demand for cosmetics in the world. Continuous development in cosmetic products and nanotechnology has led to the evolution of cosmeceuticals into nanocosmeceuticals. The use of nanotechnology in cosmetics is increasing tremendously. However, few toxic events have been reported with these nanocosmeceuticals. Thus, the safety and efficacy of these nanocosmeceuticals needs to be evaluated prior to their marketing. Various guidelines from diverse countries to regulate such products need to be harmonized. Looking at the future development and market potential, nanocosmeceuticals need more attention and awareness.

## Future perspective

Nanocosmeceuticals are expanding rapidly across the globe. Cosmeceuticals integrated with nanotechnology have shown their valuable implications in the therapy of many skin ailments. In absence of the specific guidelines, these products are undergoing fast commercialization and earning huge profits in the personal care product industry. There is an increased awareness regarding environmental concerns and risk of the side effects related to the nanoparticulate systems. Thus, there is a need for precise, harmonized guidelines and strict regulations over the future use and the advertisements of nanocosmeceuticals. Nanocosmeceuticals, a blend of cosmetics and pharmaceuticals is highly recommended for more safe and economic products for the consumers.

**Executive summary**

- Cosmetics integrated with the therapeutically active agents at the nanoscale or encapsulated in the nano-sized carriers, termed as ‘nanocosmeceuticals’ are rapidly expanding in the skin care products.
- At present, the nanocosmeceutical products are occupying a major share of cosmetic market owing to their claimed benefits.
- Various novel, sub-micron sized drug-delivery systems comprising of the lipid-based carriers, polymeric nanoparticles, nanoemulsions, inorganic nanoparticles, fullerenes and dendrimers are explored for the delivery of the cosmeceutical preparations in the skin.
- Nanocosmeceuticals exhibit different topical and transdermal mechanisms of action based on the nature, type and size of the carrier and/or actives.
- Major applications of the nanocosmeceuticals are in the sunscreens, moisturizers, hair care products, antimicrobials, anti-aging and antiwrinkle creams.
- Several scientific organizations and regulatory agencies like the US FDA and European Commission have raised the health concerns and environmental risks associated with the nanocosmeceuticals.
- Thus, evidence-based safety evaluation of the nanocosmeceuticals in context of the harmonized, globally accepted guidelines, prior to product commercialization has become essential.
